# P-101. Patient-reported outcomes and home-based self-swabs for influenza-like illness events - Lessons learned from the DANFLU-2 HomeSwab PRO Study

**DOI:** 10.1093/ofid/ofae631.308

**Published:** 2025-01-29

**Authors:** Filip Soeskov Davidovski, Kristoffer Grundtvig Skaarup, Niklas Dyrby Johansen, Daniel Modin, Nabila Shaikh, José Bartelt-Hofer, Erica Dueger, Matthew M Loiacono, Rebecca C Harris, Robertus van Aalst, Marine Dufournet, Carsten Schade Larsen, Lykke Larsen, Lothar Wiese, Michael Dalager-Pedersen, Randi Jessen, Nina Steenhard, Brian L Claggett, Scott D Solomon, Martin J Landray, Gunnar H Gislaon, Lars Køber, Pradeesh Sivapalan, Jens Ulrik Stæhr Jensen, Cyril Jean-Marie Martel, Tor Biering-Sørensen

**Affiliations:** Herlev and Gentofte Hospital, Copenhagen, Hovedstaden, Denmark; Herlev and Gentofte Hospital, Copenhagen, Hovedstaden, Denmark; Herlev and Gentofte Hospital, Copenhagen, Hovedstaden, Denmark; Copenhagen University Hospital - Herlev and Gentofte, Copenhagen, Hovedstaden, Denmark; Sanofi, Reading, England, United Kingdom; Sanofi Vaccines, Lyon France, Lyon, Rhone-Alpes, France; Sanofi Vaccines, Lyon France, Lyon, Rhone-Alpes, France; Sanofi, Reading, England, United Kingdom; Sanofi, Reading, England, United Kingdom; Sanofi Vaccines, Lyon, Auvergne, France; Sanofi, Reading, England, United Kingdom; Aarhus University Hospital, Aarhus, Midtjylland, Denmark; Odense University Hospital, Odense, Syddanmark, Denmark; Roskilde Hospital, Roskilde, Sjelland, Denmark; Aalborg University Hospital, Aalborg, Nordjylland, Denmark; SSI, Copenhagen, Hovedstaden, Denmark; SSI, Copenhagen, Hovedstaden, Denmark; Brigham and Women’s Hospital, Boston, Massachusetts; Brigham and Women’s Hospital, Harvard Medical School, Boston, Massachusetts; University of Oxford, Oxford, England, United Kingdom; Copenhagen University Hospital - Herlev and Gentofte, Copenhagen, Hovedstaden, Denmark; Copenhagen University Hospital – Rigshospitalet, Copenhagen, Hovedstaden, Denmark; Copenhagen University Hospital - Herlev and Gentofte, Copenhagen, Hovedstaden, Denmark; Copenhagen University Hospital - Herlev and Gentofte, Copenhagen, Hovedstaden, Denmark; SSI, Copenhagen, Hovedstaden, Denmark; Department of Cardiology, Herlev and Gentofte Hospital, Copenhagen, Hovedstaden, Denmark

## Abstract

**Background:**

Patient-reported outcomes (PRO) and self-swab studies may offer to improve monitoring of infectious diseases. The DANFLU-2 HomeSwab PRO study aimed to assess the practicality of self-administered swabs and digital PRO tracking to monitor influenza-like illness (ILI).

**Methods:**

As part of a sub-study of the DANFLU-2 trial, participants were instructed to self-report ILI and quality of life (QoL) using the

Respiratory Intensity and Impact Questionnaire

(RiiQ) for 14 days following symptom onset. If symptomatic, a home-based self-swab was performed, registered via a web app, and mailed to a central center for PCR testing by the participant. Weekly compliance reminders were sent through a governmental electronic letter system.

**Results:**

Overall, 1,976 DANFLU-2 participants were enrolled. During the study period, 208 participants (10.5%) filled out at least one RiiQ, among which 171 (82.2%) reported symptoms fulfilling the ILI case definition. Response rate for completing all 14 days of RiiQs following an ILI event was 85.7% [IQR: 64.3; 100]. In total, 51.4% required phone reminders due to a 48-hour non-response period within the 14 days, with a median of 1 [IQR: 1; 1] contact attempts per non-responding participant. Overall, 89.4% of participants with an ILI event reported having performed a self-swab within a median of 1 day [IQR: 0; 3] from ILI symptom onset. In total, 65.8% of swabs were correctly registered in the web app, and 96.5% of all swabs tested RNaseP positive. During the study, 36 participants (1.8%) withdrew from the study after a median of 59 days [IQR: 25; 108], primarily due to bother from weekly reminders. To reduce dropouts, the possibility of adjusting frequency of reminders was offered, with 40 participants (2.0%) opting to receive monthly reminders and 47 (2.4%) opting to receive no further reminders. The DANFLU-2 staff received 323 e-mails with participant queries about the study. Responses were given to 167 queries via e-mail and 156 via phone calls.

**Conclusion:**

The study confirmed the feasibility of using home-based self-swabs and digital PRO tracking within influenza studies. While the approach proved viable, the findings also highlighted areas for improvement in participant engagement and data collection efficiency.

**Disclosures:**

**Kristoffer Grundtvig Skaarup, MD**, Sanofi: Board Member **Nabila Shaikh, MSc**, Sanofi: Stocks/Bonds (Public Company) **José Bartelt-Hofer, Dr.**, Sanofi: Employee|Sanofi: Stocks/Bonds (Private Company) **Erica Dueger, Dr.**, Sanofi: Employee|Sanofi: Stocks/Bonds (Private Company) **Matthew M. Loiacono, PhD, MSc**, Sanofi: full-time employee|Sanofi: Stocks/Bonds (Public Company) **Rebecca C Harris, MBioch, MSc, PhD**, Sanofi vaccines: Stocks/Bonds (Private Company) **Robertus van Aalst, PhD, MSc**, Sanofi: Employee|Sanofi: Stocks/Bonds (Public Company) **Marine Dufournet, PhD**, Sanofi: Work|Sanofi: Ownership Interest **Carsten Schade Larsen, MD, DMSc**, Danske Lægers Vaccinations Service: Employee|GSK: Advisor/Consultant|GSK: Honoraria|MSD: Advisor/Consultant|MSD: Honoraria|Pfizer: Advisor/Consultant|Pfizer: Honoraria|Takeda: Advisor/Consultant|Takeda: Honoraria|Valneva: Advisor/Consultant|Valneva: Honoraria **Brian L Claggett, PhD**, Amgen: Honoraria|Cardurion: Honoraria|Corvia: Honoraria|Myokardia: Honoraria|Novartis: Honoraria **Scott D. Solomon, MD**, Actelion: Grant/Research Support|Alnylam: Grant/Research Support|Amgen: Grant/Research Support|AstraZeneca: Grant/Research Support|Bayer: Grant/Research Support|Bellerophon: Grant/Research Support|BMS: Grant/Research Support|Celladon: Grant/Research Support|Cytokintetics: Grant/Research Support|Eidos: Grant/Research Support|Gilead: Grant/Research Support|GSK: Grant/Research Support|Ionis: Grant/Research Support|Lilly: Grant/Research Support|Mesoblast: Grant/Research Support|Myokardia: Grant/Research Support|Neurotronik: Grant/Research Support|NIH/NHLBI: Grant/Research Support|Novartis: Grant/Research Support|Novartis: Honoraria|Novo Nordisk: Grant/Research Support|Sanofi: Grant/Research Support|Theracos: Grant/Research Support|US2.AI: Grant/Research Support **Lars Køber, MD, DMSc**, AstraZeneca: Honoraria|Bayer: Honoraria|Boejringer Ingelheim: Honoraria|Novartis: Honoraria|Novo Nordisk: Honoraria **Tor Biering-Sørensen, MD, PhD**, Amgen: Advisor/Consultant|Bayer: Honoraria|Boston Scientific: Honoraria|CSL Squirus: Honoraria|GE Healthcare: Honoraria|GSK: Honoraria|Novartis: Honoraria|Novo Nordisk: Honoraria|Sanofi: Honoraria

